# Effects of oral nutritional supplement on growth and body composition in malnutrition at risk and malnourished children: MARVEL study, a multi-center randomized controlled trial

**DOI:** 10.1007/s00394-026-03916-w

**Published:** 2026-05-26

**Authors:** Orapa Suteerojntrakool, Patcharapa Thaweekul, Suchaorn Saengnipanthkul, Kamolmart Wannaphahoon, Eakkarin Mekangkul, Sirinuch Chomtho

**Affiliations:** 1 Center of Excellence in Pediatric Nutrition, Department of Pediatrics, Faculty of Medicine, Chulalongkorn University, Bangkok, 10330 Thailand; 2 Ambulatory Division, Department of Pediatrics, Faculty of Medicine, Chulalongkorn University, Bangkok, 10330 Thailand; 3 Nutrition Unit, Department of Pediatrics, Faculty of Medicine, Thammasat University, Pathum Thani, 12120 Thailand; 4 Division of Nutrition, Department of Pediatrics, Faculty of Medicine, Khon Kaen University, Khon Kaen, 40002 Thailand; 5 Division of Nutrition, Department of Pediatrics, King Chulalongkorn Memorial Hospital, The Thai Red Cross Society, Bangkok, 10330 Thailand

**Keywords:** Oral nutritional supplement, Growth, Body composition, Malnutrition, Malnutrition-at-risk

## Abstract

**Purpose:**

This study investigated the effect of oral nutritional supplement (ONS) on growth and body composition in undernourished children.

**Methods:**

Thai children aged 1–6 years with weight-for-height (WFH) z-scores between − 1 SD and − 3 SD were randomized (1:1) to receive either dietary counselling alone (DC group) or counselling plus 420 mL/day of a cow’s milk–based ONS (1 kcal/mL; energy distribution: 10% protein, 49% carbohydrate, 41% fat; fortified with calcium, vitamin D, iron, and zinc) for 3 months. Weight and height were measured at baseline, 1, and 3 months and compared with WHO standards. Body composition was assessed via bioelectrical impedance analysis in children aged  ≥ 3 years old at the same period.

**Results:**

A total of 159 children (78 DC and 81 ONS), with mean ages (95% CI) of 3.5 (3.15–3.90) and 3.49 (3.10–3.90) years, were included. After 3 months, the ONS group demonstrated higher gains in weight, height, weight-for-age (WFA) and weight-for-height (WFH) z-score compared to the DC group [mean difference (95% CI): weight 0.16 (0.04–0.28) kg; height: 0.42 (0.05–0.79) cm; WFA z-score: 0.10 (0.01–0.18); WFH z-score: 0.13 (− 0.02 to 0.27)]. Gains in fat-free mass were higher with ONS at both 1 and 3 months [mean difference (95%CI): 0.26 (0.01–0.50) and 0.36 (0.10–0.63) kg]. Soft lean mass gain was also significantly greater in the ONS group at 3 months [0.39 (0.11–0.68) kg].

**Conclusions:**

ONS combined with dietary counselling improved growth and fat-free mass, supporting its role in promoting healthier body composition and long-term metabolic health. TCTR20220908004.

**Category of study:**

Randomized controlled trials.

**Supplementary Information:**

The online version contains supplementary material available at 10.1007/s00394-026-03916-w.

## Impact

Oral nutritional supplement (ONS) combined with dietary counselling represent an effective strategy to improve growth in malnutrition-at-risk and malnourished children. Beyond improving basic anthropometric measures, our study demonstrated that ONS also promoted gains in fat-free mass, which may contribute to better long-term metabolic health and physical function in undernourished children.

## Introduction

Malnutrition remains one of the most pressing public health challenges, affecting over 1600 million people worldwide [1]. Among young children, malnutrition manifests primarily as undernutrition, with the World Health Organization (WHO) reporting that approximately 149 million children under five years old suffer from stunting, while 45 million experience wasting [1].

In Thailand, malnutrition remains a significant concern. According to the latest Multiple Indicator Cluster Survey (MICS), the prevalence of underweight, stunting, and wasting among Thai children under five years of age was 8.5, 17.4, and 9.7%, respectively [2]. Alarmingly, nearly one-fifth of these children exhibited signs of severe undernutrition [2]. Additionally, findings from the 4^th^ Thailand National Health Examination Survey indicated that a substantial proportion of Thai children aged 1–5 years failed to meet their daily energy and micronutrient requirements, with deficiencies commonly observed in calcium, iron, vitamin A, and vitamin C [3]. Such nutritional gaps underscore the urgent need for effective interventions to support optimal growth and development in this vulnerable population.

Malnutrition contributes to the overall health burden such as increasing susceptibility to infectious diseases, impairing cognitive and physical development, and imposing a significant economic burden at both individual and societal levels [4–7]. Early identification of children who are malnourished or at risk is therefore essential. Previous studies have classified children “at risk” as those with an anthropometric z-score between − 1 SD and − 2 SD, indicating potential nutritional vulnerability [8]. Although no global consensus exists for this definition, it has been widely applied in research contexts, including studies from Thailand, to identify children who do not meet the criteria for malnutrition but remain vulnerable to adverse nutritional outcomes [8, 9].

Current management strategies prioritize dietary counselling, which involves educating caregivers on providing nutrient-dense foods, addressing micronutrient deficiencies, and fostering healthy eating habits tailored to the child’s developmental needs. However, multiple barriers, such as limited food access, caregiver readiness, behavioral resistance, and the complexity or variability of dietary advice, often hinder the success of dietary counselling alone [10]. Emerging evidence suggested that combining dietary counselling (DC) with oral nutritional supplement (ONS), which is an energy- and nutrient-dense formulation designed to complement regular dietary intake, might provide a more effective approach in addressing malnutrition and preventing further deterioration. Previous studies have commonly tested ONS with an energy density of 1–1.5 kcal/mL, providing balanced macronutrient distribution (protein ~ 10–15% of energy, carbohydrate 45–55%, fat 35–40%) and fortified with key micronutrients to support growth and immune function [8, 11–14].

Although several studies have demonstrated that ONS can improve weight and height gain, limited research has explored their effects on body composition, particularly lean body mass, which is directly linked to health, especially in growing children [15–17]. In this trial, the primary outcome was growth, assessed by changes in weight, with secondary outcomes including body composition. The ONS used was a newly formulated product providing balanced macronutrients and fortified essential micronutrients, especially iron, zinc, calcium, and vitamin D to support both growth and micronutrient adequacy. By evaluating the effects of this ONS in combination with dietary counselling, the study aimed to generate evidence-based insights to guide clinical practice in the management of children who are malnourished or at risk of malnutrition.

## Methods

### Study design and study population

This multicenter randomized controlled trial was a part of the MARVEL study (Malnutrition At Risk intervention for Visible and Effective Long-term growth) (TCTR 20220908004). The MARVEL study was a study designed to evaluate the effects of ONS compared with dietary counselling alone in Thai children aged 1–6 years who are malnourished or at risk of malnutrition. The study investigated a range of outcomes including growth, body composition, appetite and growth-related hormones, eating behaviors, and micronutrient status. The present trial specifically reports the effects of ONS on growth and body composition.

Data collection was conducted between September 2022 and May 2024. This study was approved by the Institutional Review Board of the Faculty of Medicine, Chulalongkorn University, COA 0717/2022, the Human Research Ethics Committee of Thammasat University, COA 228/2022 and the Khon Kaen University Ethics Committee for Human Research, HE651330.

The sample size was calculated based on Alarcon et al. [18] which reported a mean weight difference of 14.5 ± 1.5 kg vs. 13.7 ± 1.8 kg after 90 days of ONS plus DC versus DC alone. A minimum of 68 participants per group was required to achieve 80% power at a 5% significance level (two-sided). Allowing for a 15% dropout, 80 participants per group were recruited.

Children aged 1–6 years were enrolled from the outpatient department of King Chulalongkorn Memorial Hospital (Bangkok), Thammasat University Hospital (Pathum Thani) and Srinagarind Hospital (Khon Kaen), Thailand, through direct contact by researchers and social networks. The age range of 1–6 years was selected because the ONS formulation was specifically designed for children aged ≥ 1 year, and children older than 6 years were excluded to minimize the potential confounding effects of puberty and to focus on younger children in whom early nutritional intervention is most critical. Eligible participants were those with a weight-for-length/height (WFH) z-score between − 1 and − 3 SD according to the WHO Child Growth Standards, and who were born full term with a birth weight of 2.5–4.5 kg. Children with WFH < − 3 SD were excluded because severe malnutrition typically requires more intensive interventions (e.g., therapeutic feeding, micronutrient supplementation, or medical management) than dietary counselling alone in the control arm. Additional exclusion criteria included children diagnosed with conditions affecting growth or those receiving medications that interfere with appetite, nutrient absorption, or growth. Additionally, children diagnosed with cow's milk protein allergy were excluded. Informed consents were obtained from the legal guardians of all participants after a detailed explanation of the study by the research team.

### Randomization and compliance assessment

Recruited children were randomized into 2 groups using computer-generated block randomization with a block size of four. Randomization was stratified by sex to ensure balanced distribution across intervention arms. Group assignments were concealed in sealed envelopes prepared by a third party with no participant contact.

The DC group received nutritional advice from well-trained pediatricians and dietitians according to a written protocol at baseline, day 30, and day 90. Counselling was tailored to the child’s age, with recommendations based on the Thai food-based dietary guidelines for children aged 12–23 months and for those aged 2 to < 6 years. The parents or caregivers were advised on strategies to increase caloric density, such as modifying cooking methods (e.g., stir-frying or frying) or adding 1–2 teaspoons of cooking oil to meals. Additionally, caregivers were advised to promote healthy eating habits, including limiting meal duration to 30–45 minutes, maintaining a minimum interval of two hours between snacks and main meals, and serving as role models during mealtimes. This advice was consistently provided across all three study sites. Compliance with dietary advice was assessed using a 24-hour dietary recall, in which caregivers reported all foods and beverages consumed by the child in the previous day. In addition, participants were followed via phone or teleconference on days 15 and 60 to reinforce counselling messages and further evaluate adherence to dietary recommendations.

The ONS group received the same dietary advice, plus 2 servings of ONS (210 kcal/210 mL/serving) daily for 90 days. The study ONS provided balanced macronutrients (protein 10% of energy, carbohydrate 49%, fat 41%) and was fortified with micronutrients, especially calcium, vitamin D, iron, zinc (Table 1). Participants were followed on the same schedule as the DC group. Compliance with ONS intake was monitored using caregiver-maintained diary records; children who consumed at least 75% of the recommended amount, with no interruption longer than seven consecutive days, were classified as having good compliance. Adherence to dietary advice in the ONS group was assessed in the same way as the DC group, using 24-hour dietary recall at follow-up visits.

**Table 1:** Composition of the study formula^1^

Energy and nutrients	Per serving (210 mL, 45 g powder + 180 mLwater)	Unit
Energy	209.7	kcal
Energy from fat	85.4	kcal
Total protein	5.5	g
Total carbohydrates	25.6	g
Dietary fiber	1.4	g
Total fat	9.5	g
Trans fatty acids	0.05	g
Cholesterol	8.7	mg
Docosahexaenic acid DHA C22:6 (n-3)	32.9	mg
Eicosapentanoic acid EPA C20:5 (n-3)	5.9	mg
Alpha-Linolenic acid (omega-3) ALA C18:3 (n-3)	213.8	mg
*Vitamins*		
Vitamin A	101.7	mcg RE
Vitamin D	5.2	mcg
Vitamin E	2.1	mg α-TE
Vitamin K	11.3	mcg
Vitamin B1	189	mcg
Vitamin B2	257	mcg
Niacin	1.6	mg
Vitamin B6	135	mcg
Folic Acid	39.2	mcg
Vitamin B12	0.5	mcg
Vitamin C	23.3	mg
*Minerals*		
Calcium	288	mg
Phosphorus	144	mg
Magnesium	24.8	mg
Sodium	68	mg
Potassium	297	mg
Iron	3.2	mg
Zinc	1.8	mg
Copper	180	mcg
Iodine	23	mcg
Manganese	141.3	mcg
Selenium	5.4	mcg

^1^The study formula was provided /supplied by Danone Specialized Nutrition (Thailand) with expert input from the Center of Excellence in Pediatric Nutrition, Department of Pediatrics, Faculty of Medicine, Chulalongkorn University, Thailand

### Data collection

Demographic data, including age, sex, birth weight, primary caregiver, and family income, were collected through interviews conducted by research assistants. Appetite was assessed using a visual analogue scale (ranging from 1, indicating very poor appetite, to 10, indicating excellent) at baseline, on day 15, 30, 60 and 90. Also, general health information, such as defecation patterns using Bristol stool chart [19], sleep duration, and occurrences of infections, was also gathered. Additionally, adverse events, which included any illness occurring or worsening in the course of the trial, such as requiring medication for more than 14 days, the use of antibiotics, interruption of study product intake for more than 7 days or complete discontinuation, were recorded on days 15, 30, 60, and 90.

Feeding difficulties were assessed on days 0 and 90 of the study using the self-administered Montreal Children’s Hospital Feeding Scale (MCH-FS), with a T-score above 61 indicating the presence of feeding difficulties [20]. The MCH-FS has been validated in Thai children by Benjasuwantep et al., demonstrating good internal consistency (Cronbach’s alpha = 0.835) and supporting its reliability in this population [20]. All completed questionnaires were reviewed and verified by research assistants during follow-up interviews.

Energy and nutrient intakes were assessed using 24-hour dietary recalls conducted by well-trained dietitians at baseline, day 30 and day 90 of the study. The dietary data were analyzed for energy, macro- and micronutrient contents using the INMUCAL-Nutrients V. 4.0 software, developed by the Institute of Nutrition, Mahidol University, Thailand.

Anthropometric measurements, including weight, length or height, and body composition, were performed on days 0, 30, and 90 of the study by well-trained research assistants. Weight was measured to the nearest 100 g using a standard digital weighing scale (Detecto 6129, Detecto, Missouri, USA), while length was measured to the nearest 0.1 cm using a length board (Seca 417, Seca, Hamburg, Germany) or stadiometer (Detecto 6129, Detecto, Missouri, USA), depending on the child’s age. Each measurement was performed twice, with the average value used for analysis. After measurement, body mass index (BMI) was calculated using weight (kg) divided by height squared (m^2^). Additionally, weight-for-age (WFA), weight-for-length/height (WFH), length/height-for-age (HFA) z-scores and BMI z-score were determined based on the WHO Child Growth Standards using the WHO Anthro Survey Analyser [21]. Additionally, body composition was assessed in participants aged ≥ 3 years using multifrequency bioelectrical impedance analysis (BIA), in accordance with the manufacturer’s recommendations for accuracy in young children. The InBody 770 device (InBody Co., Ltd., Cerritos, CA, USA) was used at King Chulalongkorn Memorial Hospital, while the InBody 720 was used at Thammasat University Hospital and Srinagarind Hospital. BIA provided estimates of body fat mass, fat-free mass, skeletal muscle mass, percentage body fat, and total body water. Fat mass index (FMI) and fat-free mass index (FFMI) were calculated as fat mass or fat-free mass divided by height (or length) squared, in order to adjust for differences in body size [22].

### Statistical analysis

The statistical analyses were performed using Stata 18.5 (StataCorp., College Station, TX, USA). All statistical analyses were performed on an intention-to-treat basis. Before analysis, the histogram and Kolmogorov–Smirnov test was performed to evaluate statistical normality of continuous variables. Categorical variables, including sex, family income, primary caregiver, defecation pattern, infectious episodes, compliance with the ONS, and adverse events were shown as frequencies and percentages. Meanwhile, continuous data, including age, birth weight, and length, dietary intake, appetite level, anthropometry and body composition data, sleep duration, and frequency of defecation were presented as mean (95% confident interval; CI). Comparison between categorical and continuous variables between two groups were analyzed using chi-squared test and independent samples t-test, respectively. Changes in anthropometry and body composition within each group were assessed on days 0, 30, and 90 using a repeated measures ANOVA whereas the overtime changes were explored by Generalized Estimating Equation (GEE) with linear model. Further adjustment for the baseline dietary intake was done by multiple linear regression for the comparison of energy and nutrient intakes. Missing data were handled using a complete-case approach, including only participants with available data at each visit in the respective analyses. All data were analyzed according to the intention-to-treat principle. Per-protocol analyses were additionally performed among children with ≥ 75% compliance to ONS to compare outcomes between the ONS and DC groups. Subgroup analyses were also conducted based on nutritional status, stratifying participants into malnutrition-at-risk and malnourished groups. All statistical tests were 2-sided and *p* < 0.05 was considered statistically significant.

## Results

**Fig. 1:**
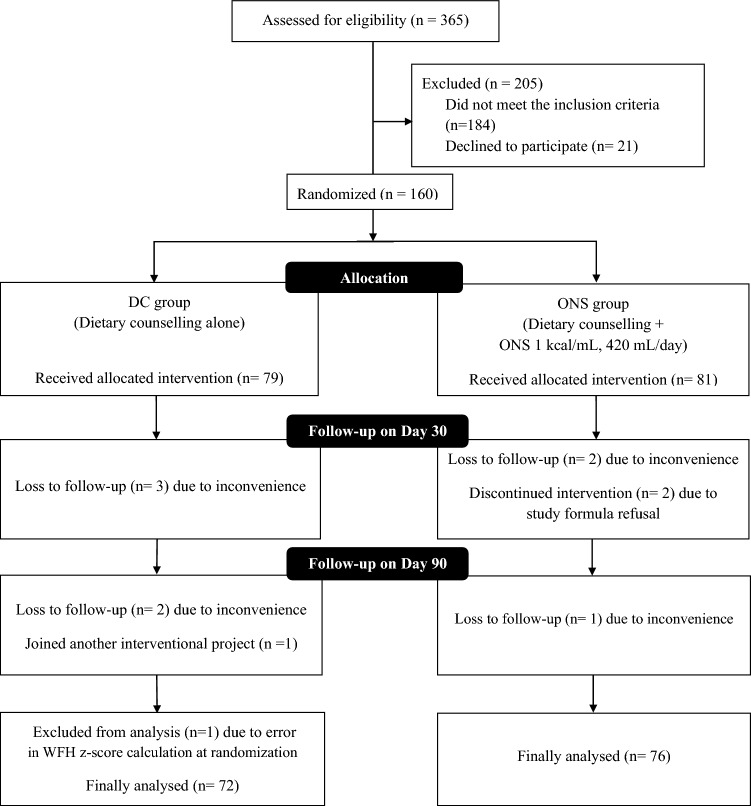
CONSORT flow diagram. DC, dietary counselling; ONS; oral nutritional supplements; WFH, weight for length or weight for height

A total of 365 eligible participants were assessed for eligibility. However, 205 children were excluded from the study: 184 did not meet the inclusion criteria, and 21 declined to participate. Consequently, 160 children were randomized into two groups: 79 in the DC group and 81 in the ONS group. During the study, 7 children from the DC group and 5 from the ONS group dropped out due to various reasons, including inconvenience to come for the follow-up visit (n = 8), refusal of the study formula (n = 2), errors in WFH z-score calculation at baseline (n = 1), and participation in another interventional project (n = 1). Consequently, on day 90, data from 72 participants in the DC group and 76 in the ONS group were analyzed. The CONSORT flow diagram of the study is shown in Fig. 1.

### Baseline characteristics

Table 2 shows baseline characteristics of study participants. The mean age (95% CI) of participants in the DC and ONS groups was 3.5 years (3.15–3.90) and 3.49 years (3.10–3.90), respectively, with nearly 60% of the participants being male. Over 60% of the children were primarily cared for by their mothers and came from middle-high socioeconomic backgrounds. One-third of the participants had no feeding difficulties, while 37% in the DC group and 40% in the ONS group were classified as having severe feeding difficulties by MCH-FS. The mean appetite level, assessed on a 10-point visual analogue scale (1 = very poor appetite to 10 = excellent appetite), was approximately 5 in both groups, with no significant difference between the DC and ONS groups. There were no significant differences between the two groups in terms of birth weight, primary caregivers, family income, appetite level, severity of feeding difficulties, or baseline anthropometry including weight, height or length and their z-score as well as body composition.

**Table 2:** Baseline characteristics of study participants (n = 159)^1^

	DC group (n = 78)	ONS group (n = 81)	*P*-value
Age (years)	3.50 (3.15–3.90)	3.49 (3.10–3.90)	0.956
Male, n (%)	46 (59)	47 (58)	0.903
Birthweight (g)	3035.9 (2970.5–3101.4)	2996.1 (2927.5–3064.8)	0.405
Main caregiver, n (%)			0.325
Mothers	52 (66.7)	49 (60.5)	
Fathers	4 (5.1)	7 (8.6)	
Grandfathers/grandmothers	10 (12.8)	17 (21)	
others	12 (15.4)	8 (9.9)	
Family income (baht/month), n (%)			0.753
< 15,000	4 (5.1)	3 (3.7)	
> 15,000–30,000	11 (14.1)	14 (17.3)	
> 30,000–50,000	23 (29.5)	30 (37.0)	
> 50,000–100,000	28 (35.9)	23 (28.4)	
> 100,000	12 (15.4)	11 (13.6)	
Feeding difficulties^2^, n (%)			0.954
No feeding difficulties	26 (33.3)	24 (29.6)	
Mild feeding difficulties	13 (16.7)	13 (16.1)	
Moderate feeding difficulties	10 (12.8)	11 (13.6)	
Severe feeding difficulties	29 (37.2)	33 (40.7)	
Appetite level^3^	4.9 (2.0–4.4)	5.4 (4.9–5.9)	0.097
Anthropometry^4^			
Weight	12.4 (11.7–13.1)	12.3 (11.5–13)	0.738
Height	94.9 (92.1–97.8)	93.9 (90.9–97)	0.632
WFA z score	− 1.58 (− 1.73 to − 1.44)	− 1.66 (− 1.82 to − 1.52)	0.494
HFA z score	− 0.85 (− 1.03 to − 0.66)	− 0.99 (− 1.22 to − 0.75)	0.342
WFH z score	− 1.67 (− 1.79 to − 1.54)	− 1.63 (− 1.76 to − 1.51)	0.712
BMI z score	− 1.61 (− 1.74 to − 1.48)	− 1.57(− 1.71 to − 1.42)	0.628
Body composition^5^			
Soft lean mass (kg)	12.1 (11.6–12.6)	11.9 (11.3–12.4)	0.529
Skeletal muscle mass (kg)	5.6 (5.3–5.9)	5.4 (5.1–5.8)	0.483
Fat-free mass (kg)	12.8 (12.2–13.3)	12.6 (12.0–13.2)	0.612
Appendicular lean mass (kg)	3.8 (3.5–4.1)	3.7 (3.4–4.0)	0.493
Body fat mass (kg)	1.5 (1.3–1.6)	1.6 (1.2–1.9)	0.579
Percent fat mass (%)	10.1 (9.0–11.2)	10.5 (8.6–12.4)	0.676
Visceral fat area (cm^2^)^6^	18.5 (16.9–20.0)	20 (18.7–21.4)	0.133
Fat mass index (kg/m^2^)	1.3 (1.2–1.5)	1.4 (1.1–1.6)	0.273
Fat free mass index (kg/m^2^)	11.7 (11.5–11.8)	11.5 (11.2–11.7)	0.735

BMI, body mass index; DC, dietary counselling; ONS, oral nutritional supplement; WFA, weight for age; HFA, height for age; WFH, weight for length or weight for height

^1^Values were presented as mean (95% CI) for continuous variables. Categorical variables were expressed as n (%). Differences in mean and proportion were tested by independent samples t-test and Chi-square test, respectively.

^2^Feeding difficulties were classified by Montreal Children’s Hospital feeding scale.

^3^Appetite level was assessed by visual analogue scale ranging from 1 (indicating low appetite) to 10 (indicating high appetite).

^4^Weight-for-age (WFA), weight-for-length/height (WFH), length/ height-for-age (HFA) z-scores and BMI z-score were determined based on the WHO Child Growth Standards using the WHO Anthro Survey Analyser.

^5^Body composition was assessed using bioelectrical impedance analysis (BIA) and measurements were performed on children aged 3 years and older (42 in the DC group and 40 in the ONS group). The fat mass index was calculated as fat mass (kg) divided by height squared (m^2^), while the fat-free mass index was computed as fat-free mass (kg) divided by height squared (m^2^).

^6^Due to measurement limitations of BIA, visceral fat area was measured in only 57 children (29 in the DC group and 28 in the ONS group).

### Compliance and acceptance of the ONS

In the ONS group, children consumed an average of 76.5% (95% CI, 50–100%) of the prescribed ONS by day 30 and 83.5% (56.9–100%) by day 90 of the study. Approximately, 60% of children in this group were classified as having good compliance according to protocol criteria (58.4% on day 30 and 61.8% on day 90). The primary reasons for ONS non-compliance were disliking the taste (40%), inconvenience in preparation (37%), and acute illness (23%).

### Energy and nutrient intakes

Table 3 presents the dietary intakes of the DC and ONS groups during the study. At randomization, energy, macronutrient, and micronutrient intakes did not differ significantly between the two groups. However, the ONS group consumed significantly more energy, fat, calcium, iron, and zinc than the DC group on both day 30 and 90. Additionally, on day 30, vitamin C and vitamin B12 intake was higher in the ONS group, though this difference was no longer significant on day 90.

**Table 3:** Comparison of dietary intakes between the DC and ONS groups at baseline, day 30 and day 90 of the study^1^

Energy and nutrient intakes	Baseline			Day 30			Day 90		
	DC group n = 78	ONS group n = 81	*P*-value	DC group n = 75	ONS group n = 77	*P*-value	DC group n = 72	ONS group n = 76	*P*-value
Energy (kcal)	986 (903.7–1068.4)	1026.1 (944–1108.3)	0.494	***1093.5 (1006–1181)***	***1240.7 (1151.5–1330)***	***0.02***	***1111.7 (1014.3–1209.1)***	***1302.4 (1194–1410.9)***	***0.01***
Protein (g)	38.2 (35–41.4)	40.5 (37.1–43.9)	0.33	44.9 (40.4–49.5)	47.9 (43.4–52.4)	0.438	44.2 (40–48.3)	48.5 (43.8–53.1)	0.173
Fat (g)	43.9 (38.3–49.6)	46.8 (41.2–52.5)	0.467	***46.1 (41.2–51.1)***	***55.8 (50.9–60.7)***	***0.007***	***44.2 (40–48.3)***	***56.4 (50.4–62.4)***	***0.035***
Vitamin A (RAE)	579.1 (371.7–786.5)	480.6 (303.6–657.7)	0.472	436.6 (300.5–572.7)	418.6 (333.8–503.4)	0.823	516.9 (259.4–774.5)	473.3 (296.7–649.9)	0.779
Vitamin B12 (mcg)	1.5 (1.1–2)	1.4 (1–1.7)	0.467	***1.4 (1.1–1.7)***	***1.8 (1.5–2.1)***	***0.04***	1.6 (1–2.1)	1.8 (1.4–2.2)	0.557
Vitamin C (mg)	40.1 (29.1–51.1)	36.8 (27.2–46.3)	0.645	***38.6 (28.9–48.4)***	***62.5 (51.1–73.9)***	***0.002***	48 (31.3–64.6)	60.7 (49.8–71.5)	0.201
Calcium (mg)	499.3 (432.7–565.9)	484.1 (416.8–551.5)	0.75	***512.5 (452.1–572.9)***	***693.5 (628.8–758.2)***	**< *0.001***	***500.5 (435.1–565.8)***	***698.6 (624.3–772.8)***	**< *0.001***
Phosphorus (mg)	558.3 (503.5–613.1)	579.6 (522.2–637.1)	0.594	627.2 (564.9–689.5)	674.5 (614.7–734.4)	0.277	619.9 (559.1–680.7)	693 (627.5–758.4)	0.091
Iron (mg)	5.8 (4.9–6.8)	5.3 (4.7–6)	0.398	***5.5 (4.8–6.2)***	***9.8 (8.6–11)***	**< *0.001***	***6 (5.2–6.9)***	***9.2 (8.4–10.1)***	**< *0.001***
Animal-based iron (mg)	3.6 (2.7–4.5)	3.4 (2.8–4)	0.731	***3.3 (2.7–3.9)***	***7.2 (6.3–8)***	**< *0.001***	***3.3 (2.8–3.9)***	***6.7 (6–7.4)***	**< *0.001***
Zinc (mg)	4.5 (3.8–5.3)	4.4 (3.8–4.9)	0.709	***4.8 (4.2–5.4)***	***6.1 (5.6–6.7)***	***0.002***	***5 (4.4–5.7)***	***6.1 (5.5–6.7)***	***0.013***

DC, dietary counselling; ONS; oral nutritional supplement

^1^Values were presented as mean (95%CI) and the differences in mean were tested by independent samples t-test. Bold and italic font indicated statistical significant at p<0.05.

After further adjustment for baseline intake, energy, fat, vitamin C, vitamin B12, calcium, iron and zinc intake remained significantly higher in the ONS group compared to the DC group (data not shown).

When intake from ONS was excluded, the ONS group had a significantly lower energy intake than the DC group on day 30 (mean difference: − 138.3 kcal/day; 95% CI: − 257.4 to − 19.1; *p* = 0.023). However, by day 90, energy intake was comparable between groups [DC group: 1111.7 kcal/day (95% CI: 1014.3–1209.1) vs. ONS group: 1019.2 kcal/day (915.1–1123.3); mean difference: − 92.5 kcal/day (− 234.2 to 49.2); *p* = 0.2]. Protein intake remained similar between the two groups throughout the study (data not shown).

### Growth and body composition

After receiving ONS for 90 days, the ONS group gained significantly more weight, height, and WFA z-score compared to the DC group [mean difference (95% CI): weight 0.16 kg (0.04–0.28), height 0.42 cm (0.05–0.79), and WFA z-score 0.10 (0.01–0.18)]. Additionally, the WFH z-score showed a trend towards improvement in the ONS group, but the difference was not statistically significant [mean difference 0.13 (− 0.02 to 0.27)] (Table 4). Moreover, subgroup analysis showed similar growth parameters among participants with WFH z-scores of − 1 to − 2 SD (malnutrition at risk) and − 2 to − 3 SD (malnourished) (Supplemental Table 1).

**Table 4:** Comparison of the changes in growth parameters from baseline to D30 and from baseline to D90 between DC and ONS groups^1,2^

	Changes from baseline							
	Day 30				Day 90			
	DC group (n = 75)	ONS group (n = 77)	Mean difference	*P*-value	DC group (n = 72)	ONS group (n = 76)	Mean difference	*P*-value
Δ weight (kg)	0.3 (0.23 to 0.37)	0.39 (0.31 to 0.48)	0.09 (− 0.02 to 0.2)	0.11	***0.52 (0.45 to 0.6)***	***0.68 (0.58 to 0.78)***	***0.16 (0.04 to 0.28)***	***0.01***
Δ WFA z score	0.08 (0.04 to 0.13)	0.15 (0.1 to 0.2)	0.07 (0 to 0.14)	0.062	***0.04 (*− *0.01 to 0.1)***	***0.14 (0.07 to 0.21)***	***0.10 (0.01 to 0.18)***	***0.03***
Δ height (cm)	0.85 (0.51 to 1.19)	0.84 (0.64 to 1.04)	− 0.01 (− 0.39 to 0.38)	0.97	***1.74 (1.49 to 1.98)***	***2.16 (1.88 to 2.43)***	***0.42 (0.05 to 0.79)***	***0.03***
Δ HFA z score	− 0.01 (− 0.07 to 0.04)	0.03 (− 0.02 to 0.09)	− 0.05 (− 0.13 to 0.03)	0.23	− 0.04 (− 0.11 to 0.02)	0.02 (− 0.06 to 0.1)	0.06 (− 0.04 to 0.16)	0.24
Δ WFH z score	0.13 (0.05 to 0.21)	0.19 (0.1 to 0.28)	0.06 (− 0.06 to 0.19)	0.30	0.08 (− 0.01 to 0.18)	0.21 (0.11 to 0.32)	0.13 (− 0.02 to 0.27)	0.08
Δ BMI z score	0.14 (0.05 to 0.23)	0.2 (0.1 to 0.3)	0.06 (− 0.08 to 0.19)	0.38	0.11 (0.01 to 0.21)	0.19 (0.08 to 0.3)	0.08 (− 0.07 to 0.23)	0.31

BMI, body mass index; DC, dietary counselling; ONS, oral nutritional supplement; WFA, weight for age; HFA, height for age; WFH, weight for length or weight for height

^1^Values were presented as mean (95% CI) and the differences in mean were tested by independent samples t-test. Bold and italic font indicated statistical significant at p<0.05.

^2^Weight-for-age (WFA), weight-for-length/height (WFH), length/height-for-age (HFA) z-scores and BMI z-score were determined based on the WHO Child Growth Standards using the WHO Anthro Survey Analyser.

**Fig. 2:**
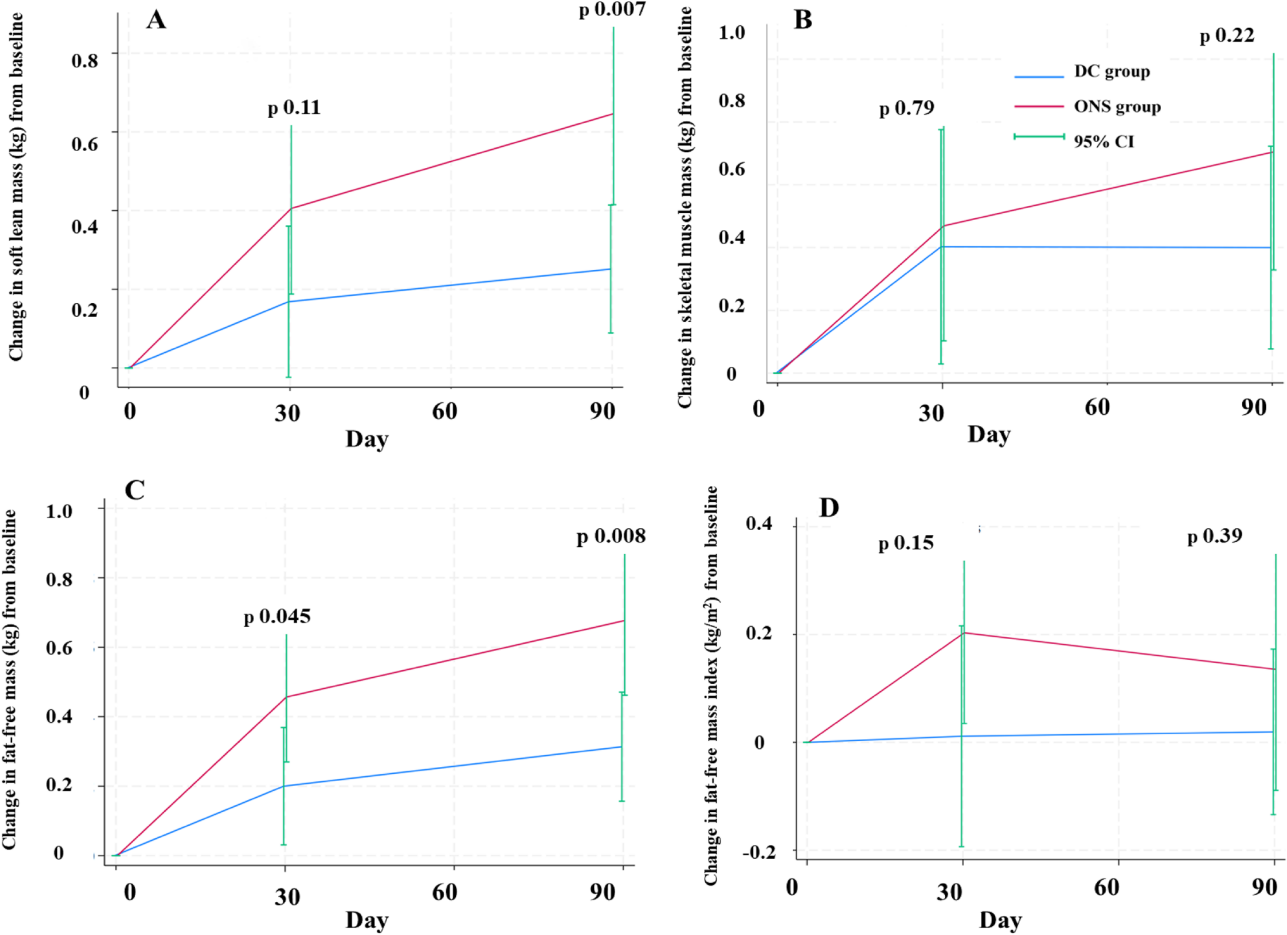
Comparison of changes in body composition assessed by bioelectrical impedance analysis among participants aged over 3 years during the 90-day study period between the DC group (n = 42) and the ONS group (n = 40). Figure 2A–D shows changes in soft lean mass (kg), skeletal mass (kg), fat-free mass (kg), and fat-free mass index (fat-free mass divided by height square, kg/m^2^), respectively. The blue and red lines represent changes in the DC and ONS groups, while the green line shows the 95% CI. Between-group comparisons were performed using independent samples t-test. DC, dietary counselling; ONS, oral nutritional supplement

Figure 2 illustrates the changes in body composition over the 90-day study period. The results indicated a significant increase in fat-free mass in the ONS group on day 30 and 90 as well as soft lean mass on day 90 [mean difference (95% CI): fat-free mass day 30: 0.26 kg (0.01–0.5), *p* = 0.04, day 90: 0.36 kg (0.10–0.63), *p* = 0.008; day 90, soft lean mass: 0.39 kg (0.11–0.68), *p* = 0.007]. Notably, the change in fat-free mass in the ONS group was significantly different from that in the DC group from day 30 onward. However, no significant differences were observed in skeletal muscle mass, fat-free mass index, fat mass, percent fat mass, fat mass index, or visceral fat mass (Supplemental Table 2).

Subgroup analyses for malnutrition-at-risk and malnourished children showed a significantly greater increase in soft lean mass and fat-free mass in the malnourished group (WFH z-scores of − 2SD to − 3SD) receiving ONS compared to those receiving dietary counselling alone at day 90 [mean changes from baseline(95%CI): soft lean mass, 0.57 (0.2–0.94) vs 0.03 (− 0.41 to 0.46) kg, *p =* 0.042 and fat-free mass, 0.62 (0.28–0.96) vs. 0.11 (− 0.3 to 0.52) kg, *p =* 0.042]. Although similar improvements were observed in the malnutrition-at-risk group (WFH z-scores of − 1SD to − 2SD), the magnitude of difference was less pronounced [mean changes from baseline (95% CI): soft lean mass, 0.69 (0.35–1.03) vs 0.35 (0.16–0.53) kg, *p =* 0.069 and fat-free mass, 0.72 (0.4–1.04) vs. 0.41 (0.23–0.59) kg, *p* = 0.083] (Supplemental Table 1).

An additional analysis was also conducted in children who complied with the intervention (consumed with ≥ 75% compliance to the prescribed ONS). The results were consistent with the primary (intention to treat) analysis. Moreover, greater improvements were observed in weight and WFA z-score, soft lean mass, fat-free mass, and fat-free mass index with significant changes detected as early as day 30 of the study (Supplemental Table 3).

Table 5 presents the changes in anthropometry and body composition within each group over the 90-day study period. Both the DC and ONS groups showed similar increases in weight, height, WFA z-score, WFH z-score, and BMI z-score on both day 30 and 90. However, improvements in soft lean mass, skeletal mass, and fat-free mass in the ONS group were evident as early as one month into the study, whereas similar changes in the DC group were observed only on day 90. In addition, a significant increase in fat-free mass index from baseline was observed only in the ONS group. In contrast, the within group change in fat mass was only apparent in the DC group on day 90.

**Table 5:** Within-group comparison of anthropometry and body composition in the DC and ONS groups over the 90-day study period^1,2^

	DC group				ONS group			
	Baseline (n = 78)	Day 30 (n = 75)	Day 90 (n = 72)	GEE	Baseline (n = 81)	Day 30 (n = 77)	Day 90 (n = 76)	GEE
Weight (kg)	12.4 (11.7 to 13.1)	***12.7 (12–13.4)***	***13 (12.3–13.7)***	***< 0.001***	12.3 (11.5–13)	***12.7 (11.9–13.4)***	***13 (12.2–13.8)***	***<0.001***
*P* value	Ref	***< 0.001***	***< 0.001***		Ref	***< 0.001***	***< 0.001***	
WFA z-score	− 1.58 (− 1.73 to to1.44)	***− 1.5 (− 1.65 to − 1.35)***	− 1.49 (− 1.64 to − 1.34)	***0.003***	− 1.66 (-1.82 to − 1.5)	***− 1.56 (− 1.72 to − 1.39)***	***− 1.55 (− 1.72 to − 1.38)***	***0.001***
*P* value	Ref	***0.002***	0.061		Ref	***0.006***	***< 0.001***	
Height (cm)	94.9 (92.1 to 97.8)	***95.4 (92.5 to 98.4)***	***96.9 (93.9 to 99.8)***	***< 0.001***	93.9 (90.9 to 97)	***94.9 (91.8 to 98)***	***96.3 (93.2 to 99.4)***	***0.001***
*P* value	Ref	***< 0.001***	***< 0.001***		Ref	***0.001***	***< 0.001***	
HFA z-score	− 0.85 (− 1.03 to − 0.66)	− 0.86 (− 1.06 to − 0.65)	− 0.83 (− 1.03 to − 0.64)	0.225	− 0.99 (− 1.23 to − 0.75)	− 1.02 (− 1.25 to − 0.78)	− 1.03 (− 1.26 to − 0.79)	0.918
*P* value	Ref	0.727	0.215		Ref	0.512	0.724	
WFH z-score	− 1.67 (− 1.79 to − 1.54)	***− 1.53 (− 1.67 to − 1.4)***	***− 1.57 (− 1.71 to − 1.42)***	***0.01***	− 1.62 (− 2.12 to − 1.24)	***− 1.44 (− 2.09 to − 1.1)***	***− 1.35 (− 1.94 to − 0.92)***	***< 0.001***
*P* value	Ref	***0.007***	***0.042***		Ref	***< 0.001***	***< 0.001***	
BMI z-score	− 1.61 (− 1.74 to − 1.48)	***− 1.47 (− 1.62 to − 1.32)***	***− 1.49 (− 1.64 to − 1.35)***	***0.004***	− 1.57 (− 1.71 to − 1.42)	***− 1.38 (− 1.56 to − 1.21)***	***− 1.37 (− 1.53 to − 1.2)***	***0.001***
*P* value	Ref	***0.006***	***0.015***		Ref	***0.001***	***0.001***	
Soft lean mass (kg)	12.1 (11.6 to 12.6)	12 (11.4 to 12.5)	***12.4 (11.9 to 12.9)***	***0.026***	11.9 (11.3 to 12.4)	***12.1 (11.5 to 12.7)***	***12.3 (11.7 to 12.9)***	*** < 0.001***
*P* value	Ref	0.226	***0.007***		Ref	***< 0.001***	***< 0.001***	
Skeletal muscle mass (kg)	5.6 (5.3 to 5.9)	5.5 (5.1 to 5.8)	***5.7 (5.4 to 6.1)***	***0.047***	5.4 (5.1 to 5.8)	***5.6 (5.2 to 6.0)***	***5.7 (5.3 to 6.0)***	***< 0.001***
*P* value	Ref	0.19	***0.015***		Ref	***0.001***	***< 0.001***	
Fat-free mass (kg)	12.8 (12.2 to 13.3)	12.7 (12 to 13.3)	***13.1 (12.6 to 13.7)***	***0.008***	12.6 (12 to 13.2)	***12.8 (12.2 to 13.5)***	***13 (12.3 to13.6)***	***< 0.001***
*P* value	Ref	0.19	***0.002***		Ref	***<0.001***	***< 0.001***	
Fat-free mass index (kg/m^2^)	11.7 (11.5 to 11.8)	11.7 (11.5 to 11.9)	11.7 (11.5 to 12)	0.797	11.5 (11.2 to 11.8)	***11.8 (11.6 to 12)***	11.6 (11.4 to 11.8)	***0.02***
*P* value	Ref	0.859	0.488		Ref	***0.006***	0.221	
Fat mass (kg)	1.46 (1.28 to 1.65)	1.5 (1.29 to 1.72)	***1.69 (1.42 to 1.96)***	0.128	1.57 (1.22 to 1.92)	1.45 (1.2 to 1.7)	1.88 (1.26 to 2.51)	0.225
*P* value	Ref	0.513	***0.042***		Ref	0.987	0.142	
Percent fat mass (%)	10.1 (9.0 to 11.2)	10.5 (9.1 to 11.9)	10.5 (9.3 to 11.7)	0.645	10.5 (8.6 to 12.4)	9.9 (8.4 to 11.4)	11 (9.4 to12.5)	0.382
*P* value	Ref	0.388	0.462		Ref	0.354	0.696	
Visceral fat area (cm^2^)	18.5 (16.9 to 20)	18.7 (17.3 to 20.2)	18.6 (17.2 to 19.9)	0.479	20 (18.7 to 21.4)	19 (17.4 to 20.5)	19.2 (17.9 to 20.5)	0.425
*P* value	Ref	0.873	0.299		Ref	0.209	0.336	
Fat-mass index (kg/m^2^)	1.3 (1.2 to 1.5)	1.4 (1.2 to 1.6)	1.5 (1.3 to 1.8)	0.244	1.36 (1.12 to 1.61)	1.3 (1.1 to 1.51)	1.6 (1.17 to 2.03)	0.252
*P* value	Ref	0.578	0.079		Ref	0.958	0.166	

BMI, body mass index; DC, dietary counselling; ONS, oral nutritional supplement; GEE, Generalized Estimating Equation; Ref, reference; WFA, weight for age; HFA, height for age; WFH, weight for length or weight for height

^1^The data were presented as mean (95% CI). Comparison of the changes within groups were analyzed using repeated measures ANOVA, while trends over time within groups were assessed using Generalized Estimating Equation. Bold and italic font indicated statistical significant at p<0.05.

^2^Weight-for-age (WFA), weight-for-length/height (WFH), length/ height-for-age (HFA) z-scores and BMI z-score were determined based on the WHO Child Growth Standards using the WHO Anthro Survey Analyser. Fat mass index and fat-free mass index were calculated as fat mass or fat-free mass divided by height (or length) squared.

### General health and adverse events

Sleep duration, including both daytime and nighttime sleep, was similar between the DC and ONS groups. On day 90, the mean (95% CI) sleep duration in hours was as follows: daytime sleep 1.60 (1.30–1.90) in the DC group vs. 1.47 (1.24–1.70) in the ONS group; nighttime sleep 9.53 (9.18–9.88) vs. 9.63 (9.41–9.86), respectively. Defecation frequency and stool patterns, as assessed by the Bristol Stool Chart, were also comparable between the two groups on day 30 and 90. No significant difference in infection frequencies were observed between the two groups at any time-point (Supplemental Table 4). Appetite levels improved in both the DC and ONS groups on day 90, but there were no significant differences between the two groups [on day 90: DC group, 6.4 (5.9–6.9); ONS group 6.6 (6.1–6.9), *p* = 0.665].

A total of six serious adverse events were reported: five in the ONS group and one in the DC group. None were related to the intervention. In the ONS group, the hospital admissions were due to RSV infection (n = 1), salmonella gastroenteritis (n = 1), acute bronchitis and gastroenteritis (n = 1), dengue infection (n = 1), and pneumonia of unidentified etiology (n = 1). In the DC group, one participant was hospitalized due to RSV pneumonia. However, there were no statistically significant differences in the adverse events between the two groups.

## Discussion

Malnutrition remains a significant health concern among children worldwide, prompting various nutritional interventions aimed at addressing this issue. This study demonstrated that dietary counselling combined with ONS is an effective strategy to improve growth parameters particularly weight, height, WFA z-score and body composition, with notable increases in fat-free mass.

Our study found that both malnutrition-at-risk and malnourished children showed improvements in weight and height over the 90-day study period, whether they received dietary counselling alone or in combination with ONS. However, children in the ONS group experienced significantly greater gains in weight, height, and WFA z-scores compared to those who received dietary counselling alone. These findings align with a meta-analysis by Zhang et al., which reported that children with a mild, moderate or severe degree of undernutrition receiving ONS for 90 days achieved greater gains of 0.199 (95% CI 0.099 − 0.297) WFA z-score, 0.053 (0.018–0.088) in HFA z-score and 0.277 (0.205–0.349) in WFH z-score compared to those obtaining dietary advice alone [16]. Similarly, a recent randomized controlled trial by Ow et al. in Vietnam, involving children with WFA and HFA z-scores below -1 SD, demonstrated that those who received ONS in combination with dietary counselling showed significantly greater gains by day 30, including a 0.21 kg increase in weight, a 0.12 increase in WFA z-score, and a 0.16 increase in WFH z-score, compared to those who received counselling alone. [15] In comparison, our study demonstrated smaller improvements in WFA, HFA, and WFH z-scores. This may be attributed to differences in recruitment criteria, the thoroughness of dietary counselling, and varying levels of compliance with dietary advice or ONS intake. In our subgroup analysis, children who consumed more than 75% of the prescribed ONS showed significantly greater improvements in weight and WFA z-scores on day 30 compared to the DC group. Our findings were consistent with previous studies that had reported early improvements in weight-related parameters with ONS supplementation [14, 15]. These findings further support the effectiveness of combining ONS with dietary counselling as a strategy to enhance growth outcomes in malnutrition-at-risk and malnourished children.

However, despite a significant increase in height, our study found no significant difference in HFA z-score changes observed between the ONS and DC groups. In contrast, a previous meta-analysis found significant improvements in both height and HFA z-scores after 90 days of ONS intervention compared to dietary advice [16]. This discrepancy may be due to the longer duration typically required for linear growth to be reflected in HFA z-scores, as linear growth responds more slowly to nutritional interventions than weight gain. Similar observations were noted in a study by Ow et al. [15] where height differences became significant only on day 120, while interim measurements were unavailable on day 90. This may be attributed to the longer duration required to observe meaningful changes in linear growth. Therefore, future studies with a longer follow-up period are recommended to better assess the impact of ONS on linear growth and HFA z-scores.

Additionally, subgroup analysis revealed that malnutrition-at-risk children who received ONS experienced improvements in growth comparable to those observed in malnourished children, although the magnitude of these changes was less pronounced. These findings are consistent with previous research suggesting that ONS, when combined with dietary advice, can enhance growth outcomes in children with suboptimal nutritional status. For example, Huynh et al. [14] conducted a study in Filipino children aged 3–4 years with weight-for-height percentiles between the 5^th^ and 25^th^ and found that ONS supplementation led to the greatest increase in weight-for-height percentiles within the first 4 weeks, while height-for-age percentiles increased steadily and became significantly higher than baseline from week 24 onward. Similarly, a study in India also found that picky-eating children with WFH percentiles between the 5^th^ and 25^th^ were able to meet their nutritional requirements after receiving ONS, without disrupting their normal food consumption patterns [23]. These findings indicated that ONS was effective not only in treating malnourished children but also in preventing its progression among at-risk populations.

Interestingly, in addition to similar findings of improvement in weight and height gain, our study found that the addition of ONS to dietary counselling enhanced soft lean mass, skeletal muscle mass, and fat-free mass. Notably, the positive effects on lean mass remained evident in the ONS group even after adjusting for height using the fat-free mass index. This finding indicated that the greater fat-free mass gain did not simply result from the greater increase in height. Additionally, the increase in fat-free mass in the ONS group can be observed as early as day 30 of the study. A similar finding was reported in adults by Jayawardena et al. [17] who studied older adults with or at risk of malnutrition and showed that participants receiving ONS had greater increases in lean mass, as assessed by dual-energy X-ray absorptiometry, compared to those who received dietary counselling alone. The SPROUT study, conducted in children with or at risk of malnutrition, found that those who received ONS alongside dietary counselling had significantly greater mid-upper-arm muscle circumference and arm muscle area compared to the DC group, supporting our findings [15]. However, there has been no prior study measuring the changes in body composition in growing young children after ONS supplementation as in our study. A possible explanation for the effect of ONS on enhancing fat-free mass is that it provides a consistent, concentrated source of high-quality protein, energy, and essential micronutrients that support muscle and other protein synthesis. Increasing lean body mass may also offer greater long-term health benefits than weight gain alone, as it contributes to improved metabolic health, physical function, and overall growth quality, rather than simply increasing fat mass [24, 25].

Although this study showed higher intakes of energy, fat, and micronutrients, including vitamins B12 and C, calcium, iron, and zinc in the ONS group compared to the DC group, protein intake did not differ significantly between the two groups. This suggested that the observed improvements in anthropometric measures and body composition may be attributed more to the increased overall energy and micronutrient intakes rather than protein alone. In our study, both groups already met their protein requirements according to the Dietary Reference Intake (DRI) at baseline; therefore, the additional energy and nutrient density provided by ONS may have contributed to the observed differences. Beyond energy and protein, several micronutrients, such as zinc, iron, vitamin B12, and vitamin D, play critical roles in muscle mass development [26–30]. A systematic review examining the impact of zinc supplementation on body composition in children indicated that zinc may improve fat-free mass, particularly in those with existing growth failure [26]. However, the current evidence remains limited due to the small number of studies and variability in design. Zinc is essential for lean tissue synthesis, and its deficiency has been associated with increased energy cost of tissue deposition. Iron also appears to influence muscle development. Vinke et al. [27] reported that iron deficiency may impair muscle mass by disrupting myoblast proliferation, reducing aerobic glycolytic capacity, and increasing markers of myocyte atrophy and apoptosis. In addition, a study from China found a positive correlation between serum vitamin B12 levels and both total and appendicular lean mass, further supporting the role of micronutrients in body composition regulation [28]. Moreover, emerging evidence also indicated that vitamin D contributed to skeletal muscle maintenance through vitamin D receptor–mediated regulation of mitochondrial function, oxidative stress, and satellite cell activity, suggesting that adequate vitamin D status may support muscle mass preservation and accretion, particularly in populations at risk of deficiency [29]. Likewise, a South African study also demonstrated that 9 months of multiple micronutrient supplementation significantly increased fat-free mass in school-aged children [30]. Together, these findings highlight the importance of comprehensive nutritional support including key vitamins and trace elements in promoting healthy growth and lean mass development in malnourished and malnutrition at risk children, beyond macronutrient intake alone.

Even though dietary counselling is the standard approach for managing malnutrition in children, its effectiveness can be limited by the variability in dietary intake provided by caregivers and the differing levels of expertise among counsellors. Elhady et al. [10] reported that barriers to delivering adequate nutritional care to malnourished children in low-resource settings included healthcare-related factors such as limited nutrition education, an insufficient number of healthcare providers, and inadequate time allocated per patient, as well as cultural factors and prevailing nutritional beliefs. By comparison, the use of ONS combined with dietary advice may offer a more practical and consistent strategy, particularly in clinical settings. ONS provides a reliable source of essential nutrients, especially for children with poor appetite or feeding difficulties, ensuring more reliable nutrient intake. On the other hand, the long-term cost of the ONS vs. comprehensive dietary counselling by experienced specialists is also an important aspect to consider. In resource limiting settings, the efforts should be put in place both for improving diet quality and feeding behavior as a long-term strategy while additional ONS during the initial phase, at least for the first 3-month, could boost the recovery of growth and improve body composition.

A key strength of this study lied in its comprehensive assessment approach. No prior data reported body composition changes after the intervention with ONS in growing children. We measured body composition and demonstrated that ONS can lead to the increase in lean body mass. Furthermore, we compared energy intake as well as macro- and micronutrient consumption between groups, providing a more in-depth understanding into the micronutrient aspect related to changes in growth and body composition. However, several limitations should be acknowledged. Firstly, body composition was assessed using bioelectrical impedance analysis, which may affect the validity of measurements and was only applicable in children older than three years. Secondly, the short duration of follow-up limits the ability to assess the long-term effects of ONS on growth and body composition. Future research should consider using more precise methods for assessing body composition, such as dual-energy X-ray absorptiometry, and extending the follow-up period to evaluate the sustainability of ONS benefits over time.

## Conclusion

This study demonstrated that ONS combined with dietary counselling was effective in improving growth and lean body mass in malnourished and malnutrition at-risk children. These findings highlight the clinical value of incorporating ONS into nutritional management strategies to enhance fat-free mass which may associate with better metabolic health and physical function. While short-term benefits are evident, further research with longer follow-up and mechanistic exploration is warranted to confirm the sustained impact and underlying pathways of the ONS intervention.

## Supplementary Information

Below is the link to the electronic supplementary material.

Supplementary file1 (DOCX 42 KB)

## Data Availability

Data described in the manuscript, codebook, and analytic code will be made available upon reasonable request in a de-identified form, in compliance with ethical guidelines and with the ethics committee’s approval.
