# Supplementary material for: Effects of oral nutritional supplement on growth and body composition in malnutrition at risk and malnourished children: MARVEL study, a multi-center randomized controlled trial
Source: Eur J Nutr. 2026 May 26;65(4):135. doi: 10.1007/s00394-026-03916-w (PMC13212650; doi:10.1007/s00394-026-03916-w)
Supplement: Supplementary file 1 — Supplementary file1 (DOCX 42 KB) [file 394_2026_3916_MOESM1_ESM.docx]

**Supplemental Table 1** Comparison of the changes in anthropometry and body composition between the DC group and ONS group, divided into subgroups of malnutrition-at-risk and malnourished children^1,2^

|  | **Changes from baseline to day 30** | | | **Changes from baseline to day 90** | | |
| --- | --- | --- | --- | --- | --- | --- |
| **Malnutrition-at-risk children**  **(WFH z-score between -1SD to -2SD)** | **DC group (n= 54)** | **ONS group (n= 46)** | **P-value** | **DC group (n= 52)** | **ONS group (n= 46)** | **P-value** |
| **∆Weight (kg)** | ***0.3 (0.22 to 0.38)*** | ***0.44 (0.32 to 0.56)*** | ***0.047*** | ***0.53 (0.44 to 0.62)*** | ***0.72 (0.6 to 0.85)*** | ***0.012*** |
| **∆WFA z-score** | ***0.08 (0.02 to 0.13)*** | ***0.17 (0.1 to 0.24)*** | ***0.036*** | ***0.04 (-0.03 to 0.1)*** | ***0.16 (0.08 to 0.24)*** | ***0.016*** |
| **∆Height (cm)** | 0.85 (0.39 to 1.31) | 0.74 (0.49 to 0.98) | 0.686 | ***1.75 (1.48 to 2.02)*** | ***2.21 (1.83 to 2.59)*** | ***0.047*** |
| **∆HFA z-score** | -0.02 (-0.08 to 0.04) | -0.01 (-0.07 to 0.06) | 0.778 | -0.05 (-0.12 to 0.03) | 0.03 (-0.08 to 0.14) | 0.228 |
| **∆WFH z score** | 0.13 (0.03 to 0.22) | 0.25 (0.14 to 0.36) | 0.097 | 0.09 (-0.03 to 0.21) | 0.24 (0.11 to 0.37) | 0.097 |
| **∆BMI z score** | 0.13 (0.03 to 0.24) | 0.26 (0.14 to 0.38) | 0.113 | 0.1 (-0.03 to 0.23) | 0.2 (0.06 to 0.34) | 0.281 |
| **∆Soft lean mass (kg)** | 0.22 (-0.01 to 0.46) | 0.41 (0.1 to 0.72) | 0.309 | 0.35 (0.16 to 0.53) | 0.69 (0.35 to 1.03) | 0.069 |
| **∆Skeletal muscle mass (kg)** | 0.4 (-0.03 to 0.84) | 0.58 (0.03 to 1.13) | 0.594 | 0.41 (0.05 to 0.77) | 0.76 (0.25 to 1.26) | 0.248 |
| **∆Fat-free mass (kg)** | 0.25 (0.04 to 0.45) | 0.5 (0.24 to 0.75) | 0.120 | 0.41 (0.23 to 0.59) | 0.72 (0.4 to 1.04) | 0.083 |
| **∆Fat-free mass index (kg/m^2^)** | 0.03 (-0.21 to 0.27) | 0.26 (0.03 to 0.49) | 0.167 | 0.02 (-0.15 to 0.2) | 0.19 (-0.11 to 0.5) | 0.315 |
| **∆Fat mass (kg)** | 0.1 (-0.1 to 0.31) | 0.15 (-0.11 to 0.41) | 0.762 | 0.1 (-0.06 to 0.26) | 0.6 (-0.1 to 1.31) | 0.144 |
| **∆Percent fat mass (%)** | 0.65 (-0.92 to 2.22) | 0.24 (-1.23 to 1.71) | 0.704 | 0.55 (-0.58 to 1.68) | 0.26 (-1.8 to 2.32) | 0.797 |
| **∆Visceral fat area (cm^2^)** | 1.88 (-0.49 to 4.25) | 0.26 (-1.3 to 1.82) | 0.267 | 0.59 (-1.3 to 2.48) | 0.09 (-1.41 to 1.59) | 0.677 |
| **∆Fat-mass index (kg/m^2^)** | 0.13 (-0.08 to 0.35) | 0.14 (-0.1 to 0.39) | 0.953 | 0.12 (-0.06 to 0.31) | 0.17 (-0.14 to 0.49) | 0.785 |
| **Malnourished children**  **(WFH z-score between -2SD to -3SD)** | **DC group (n= 15)** | **ONS group (n=19)** | **P-value** | **DC group (n=14)** | **ONS group (n=17)** | **P-value** |
| **∆Weight (kg)** | 0.3 (0.08- 0.52) | 0.32 (0.16 to 0.48) | 0.867 | 0.46 (0.28 to 0.64) | 0.6 (0.38 to 0.82) | 0.302 |
| **∆WFA z-score** | 0.11 (-0.02 to 0.24) | 0.13 (0.02 to 0.25) | 0.782 | 0.03 (-0.09 to 0.15) | 0.13 (-0.04 to 0.3) | 0.354 |
| **∆Height (cm)** | 0.78 (0.36 to 1.19) | 1.08 (0.66 to 1.5) | 0.292 | 1.48 (0.82 to 2.15) | 1.99 (1.36 to 2.62) | 0.250 |
| **∆HFA z-score** | -0.02 (-0.18 to 0.15) | 0.11 (-0.01 to 0.22) | 0.178 | -0.08 (-0.24 to 0.08) | -0.04 (-0.24 to 0.15) | 0.761 |
| **∆WFH z score** | 0.16 (-0.05 to 0.37) | 0.1 (-0.07 to 0.28) | 0.656 | 0.03 (-0.2 to 0.26) | 0.22 (-0.02 to 0.46) | 0.232 |
| **∆BMI z score** | 0.17 (-0.07 to 0.42) | 0.1 (-0.09 to 0.29) | 0.628 | 0.13 (-0.07 to 0.33) | 0.23 (-0.03 to 0.49) | 0.527 |
| **∆Soft lean mass (kg)** | -0.04 (-0.52 to 0.44) | 0.37 (-0.02 to 0.75) | 0.144 | ***0.03 (-0.41 to 0.46)*** | ***0.57 (0.2 to 0.94)*** | ***0.042*** |
| **∆Skeletal muscle mass (kg)** | 0.44 (-0.65 to 1.54) | 0.2 (-0.01 to 0.41) | 0.619 | 0.48 (-0.55 to 1.5) | 0.7 (-0.19 to 1.59) | 0.713 |
| **∆Fat-free mass (kg)** | 0.01 (-0.41 to 0.44) | 0.39 (-0.01 to 0.79) | 0.152 | ***0.11 (-0.3 to 0.52)*** | ***0.62 (0.28 to 0.96)*** | ***0.042*** |
| **∆Fat-free mass index (kg/m^2^)** | -0.16 (-0.72 to 0.39) | -0.01 (-0.27 to 0.25) | 0.549 | -0.01 (-0.49 to 0.46) | 0.04 (-0.46 to 0.54) | 0.858 |
| **∆Fat mass (kg)** | 0.31 (0.06 to 0.56) | -0.11 (-0.62 to 0.4) | 0.106 | 0.36 (0.02 to 0.69) | 0.11 (-0.31 to 0.52) | 0.327 |
| **∆Percent fat mass (%)** | 1.98 (0.31 to 3.64) | -0.73 (-3.91 to 2.44) | 0.100 | 2.17 (0.02 to 4.31) | 1.04 (-1.95 to 4.02) | 0.516 |
| **∆Visceral fat area (cm^2^)** | 2.41 (-4.57 to 9.39) | 0.72 (-0.36 to 1.8) | 0.589 | 2.48 (-4.14 to 9.09) | 1.93 (-1.8 to 5.65) | 0.864 |
| **∆Fat-mass index (kg/m^2^)** | 0.26 (0.05 to 0.48) | -0.08 (-0.51 to 0.34) | 0.114 | 0.31 (0.04 to 0.58) | 0.12 (-0.28 to 0.51) | 0.400 |

Abbreviation: BMI, body mass index; DC, dietary counselling; ref, reference; ONS, oral nutritional supplement; WFA, weight for age; HFA, height for age; WFH, weight for length or weight for height

^1^Values were presented as mean (95%CI) and the differences in mean were tested by independent samples t-test.

^2^Weight-for-age (WFA), weight-for-length/height (WFH), length/ height-for-age (HFA) z-scores and BMI z-score were determined based on the WHO Child Growth Standards using the WHO Anthro Survey Analyser. Fat mass index and fat-free mass index were calculated as fat mass or fat-free mass divided by height (or length) squared.

|  | **Changes from baseline** | | | | | | | |
| --- | --- | --- | --- | --- | --- | --- | --- | --- |
|  | **Day 30** | | | | **Day 90** | | | |
|  | **DC group**  **n= 42** | **ONS group**  **n= 39** | **Mean difference** | **P-value** | **DC group**  **n= 40** | **ONS group**  **n= 39** | **Mean difference** | **P-value** |
| **Δ Soft lean mass (kg)** | 0.17  (-0.02 to 0.36) | 0.41  (0.19 to 0.62) | 0.24  (-0.05 to 0.52) | 0.11 | ***0.25***  ***(0.09 to 0.41)*** | ***0.65***  ***(0.42 to 0.88)*** | ***0.39***  ***(0.11 to 0.68)*** | ***0.007*** |
| **ΔSkeletal muscle mass(kg)** | 0.4  (0.03 to 0.78) | 0.47  (0.1 to 0.84) | 0.07  (-0.45 to 0.58) | 0.79 | 0.4  (0.08 to 0.72) | 0.7  (0.33 to 1.08) | 0.3  (-0.19 to 0.8) | 0.22 |
| **Δ Fat-free mass (kg)** | ***0.2***  ***(0.03 to 0.37)*** | ***0.46***  ***(0.27 to 0.64)*** | ***0.26***  ***(0.01 to 0.5)*** | ***0.04*** | ***0.31***  ***(0.16 to 0.47)*** | ***0.68***  ***(0.46 to 0.89)*** | ***0.36***  ***(0.1 to 0.63)*** | ***0.008*** |
| **Δ Appendicular lean mass (kg)** | -0.3  (-1.13 to 0.53) | 0.24  (0.02 to 0.47) | 0.54  (-0.32 to 1.4) | 0.21 | -0.41  (-1.29 to 0.46) | 0.28  (0.01 to 0.54) | 0.69  (-0.18 to 1.56) | 0.12 |
| **Δ Body fat mass (kg)** | 0.16  (0 to 0.32) | 0.09  (-0.13 to 0.31) | -0.07  (-0.34 to 0.19) | 0.59 | 0.2  (0.05 to 0.34) | 0.44  (-0.01 to 0.89) | 0.24  (-0.24 to 0.72) | 0.32 |
| **Δ Percent fat mass (%)** | 0.99  (-0.19 to 2.17) | -0.31  (-1.54 to 0.93) | -1.3  (-2.98 to 0.39) | 0.12 | 1.15  (0.16 to 2.13) | 0.33  (-1.17 to 1.83) | -0.82  (-2.59 to 0.96) | 0.36 |
| **Δ Visceral fat area (cm^2^)** | 1.89  (-0.24 to 4.02) | 0.31  (-0.94 to1.56) | -1.58  (-4.04 to 0.89) | 0.21 | 0.92  (-0.93 to 2.77) | 0.81  (-0.59 to 2.21) | -0.11  (-2.37 to 2.15) | 0.93 |
| **Δ Fat-free mass index (kg/m^2^)** | 0.01  (-0.19 to 0.22) | 0.2  (0.03 to 0.37) | 0.19  (-0.07 to 0.45) | 0.15 | 0.02  (-0.13 to 0.17) | 0.14  (-0.09 to 0.36) | 0.12  (-0.15 to 0.39) | 0.39 |
| **Δ Fat-mass index (kg/m^2^)** | 0.17  (0.01 to 0.33) | 0.09  (-0.13 to 0.3) | -0.08  (-0.34 to 0.18) | 0.54 | 0.19  (0.04 to 0.35) | 0.16  (-0.07 to 0.39) | -0.03  (-0.31 to 0.24) | 0.82 |

**Supplemental Table 2** Comparison of changes in body composition assessed by bioelectrical impedance analysis among participants aged over 3 years during the 90-day study period between the DC group and the ONS group

Abbreviation: BMI, body mass index; DC, dietary counselling; ref, reference; ONS, oral nutritional supplement

^1^Values were presented as mean (95%CI) and the differences in mean were tested by independent samples t-test

**Supplemental Table 3** Comparison of changes in growth and body composition between DC group and ONS group with ≥75% compliance to prescribed ONS^1,2^

|  | **Changes from baseline to day 30** | | | | **Changes from baseline to day 90** | | | |
| --- | --- | --- | --- | --- | --- | --- | --- | --- |
|  | **DC group (n=75)** | **ONS group (n=45)** | **Difference** | **P-value** | **DC group (n=73)** | **ONS group (n=50)** | **Difference** | **P-value** |
| **Δ Weight (kg)** | ***0.3***  ***(0.23 to 0.37)*** | ***0.47***  ***(0.34 to 0.6)*** | ***0.17***  ***(0.03 to 0.3)*** | ***0.01*** | ***0.52***  ***(0.45 to 0.6)*** | ***0.72***  ***(0.6 to 0.85)*** | ***0.2***  ***(0.07 to 0.33)*** | ***<0.001*** |
| **Δ WFA z score** | ***0.08***  ***(0.04 to 0.13)*** | ***0.18***  ***(0.11 to 0.26)*** | ***0.1***  ***(0.02 to 0.18)*** | ***0.02*** | ***0.04***  ***(-0.01 to 0.1)*** | ***0.18***  ***(0.1 to 0.25)*** | ***0.13***  ***(0.04 to 0.22)*** | ***<0.001*** |
| **Δ height (cm)** | 0.85  (0.51 to 1.19) | 0.83  (0.57 to 1.1) | -0.01  (-0.5 to 0.47) | 0.95 | 1.74  (1.49 to 1.98) | 2.11  (1.77 to 2.46) | 0.38  (-0.03 to 0.79) | 0.07 |
| **Δ HFA z score** | -0.01  (-0.07 to 0.04) | 0.04  (-0.03 to 0.11) | 0.05  (-0.04 to 0.14) | 0.27 | -0.04  (-0.11 to 0.02) | 0.02  (-0.07 to 0.11) | 0.06  (-0.04 to 0.17) | 0.23 |
| **Δ WFH z score** | 0.13  (0.05 to 0.21) | 0.24  (0.12 to 0.36) | 0.11  (-0.03 to 0.25) | 0.13 | 0.08  (-0.01 to 0.18) | 0.23  (0.1 to 0.36) | 0.15  (-0.01 to 0.31) | 0.07 |
| **Δ BMI z score** | 0.14  (0.05 to 0.23) | 0.25  (0.11 to 0.38) | 0.11  (-0.05 to 0.26) | 0.17 | 0.11  (0.01 to 0.21) | 0.24  (0.09 to 0.38) | 0.13  (-0.04 to 0.3) | 0.13 |
| **Δ Soft lean mass (kg)** | ***0.17***  ***(-0.02 to 0.36)*** | ***0.63***  ***(0.44 to 0.82)*** | ***0.46***  ***(0.17 to 0.76)*** | ***<0.001*** | ***0.25***  ***(0.09 to 0.41)*** | ***0.7***  ***(0.38 to 1.02)*** | ***0.45***  ***(0.12 to 0.78)*** | ***0.01*** |
| **Δ Skeletal muscle mass (kg)** | 0.4  (0.03 to 0.78) | 0.79  (0.2 to 1.37) | 0.38  (-0.27 to 1.04) | 0.24 | 0.4  (0.08 to 0.72) | 0.44  (0.22 to 0.65) | 0.04  (-0.38 to 0.46) | 0.85 |
| **Δ Fat-free mass (kg)** | ***0.2***  ***(0.03 to 0.37)*** | ***0.67***  ***(0.47 to 0.87)*** | ***0.47***  ***(0.2 to 0.74)*** | ***<0.001*** | ***0.31***  ***(0.16 to 0.47)*** | ***0.71***  ***(0.42 to 1.01)*** | ***0.4***  ***(0.09 to 0.7)*** | ***0.01*** |
| **Δ Fat-free mass index (kg/m^2^)** | ***0.01***  ***(-0.19 to 0.22)*** | ***0.33***  ***(0.16 to 0.49)*** | ***0.31***  ***(0.01 to 0.62)*** | ***0.04*** | 0.02  (-0.13 to 0.17) | 0.15  (-0.16 to 0.47) | 0.13  (-0.18 to 0.45) | 0.4 |
| **Δ Fat mass (kg)** | 0.16  (0 to 0.32) | 0.09  (-0.26 to 0.44) | -0.07  (-0.4 to 0.26) | 0.67 | 0.2  (0.05 to 0.34) | 0.46  (-0.18 to 1.09) | 0.26  (-0.3 to 0.81) | 0.36 |
| **Δ Percent fat mass (%)** | 0.99  (-0.19 to 2.17) | -0.88  (-2.44 to 0.68) | -1.87  (-3.85 to 0.1) | 0.06 | 1.15  (0.16 to 2.13) | -0.04  (-2.06 to 1.99) | -1.18  (-3.21 to 0.84) | 0.25 |
| **Δ Visceral fat area (cm^2^)** | 1.89  (-0.24 to 4.02) | 0.9  (-1.02 to 2.83) | -0.98  (-4.13 to 2.17) | 0.53 | 0.92  (-0.93 to 2.77) | -0.09  (-1.3 to 1.13) | -1.01  (-3.4 to 1.38) | 0.4 |
| **Δ fat-mass index (kg/m^2^)** | 0.17  (0.01 to 0.33) | 0.1  (-0.23 to 0.43) | -0.07  (-0.38 to 0.25) | 0.68 | 0.19  (0.04 to 0.35) | 0.09  (-0.2 to 0.37) | -0.11  (-0.4 to 0.19) | 0.47 |

Abbreviation: BMI, body mass index; DC, dietary counselling; ref, reference; ONS, oral nutritional supplement WFA, weight for age; HFA, height for age; WFH, weight for length or weight for height

^1^Values were presented as mean (95%CI) and the differences in mean were tested by independent samples t-test.

^2^Weight-for-age (WFA), weight-for-length/height (WFH), length/ height-for-age (HFA) z-scores and BMI z-score were determined based on the WHO Child Growth Standards using the WHO Anthro Survey Analyser.

**Supplemental Table 4** Comparison of sleep duration, defecation and infectious episodes between DC and ONS group^1^

|  | **Day 15** | | **Day 30** | | **Day 60** | | **Day 90** | |
| --- | --- | --- | --- | --- | --- | --- | --- | --- |
|  | **DC group** | **ONS group** | **DC group** | **ONS group** | **DC group** | **ONS group** | **DC group** | **ONS group** |
| **Sleep duration** | | | | | | | | |
| Daytime sleep (hours) | 2.61 (1.25-1.95) | 1.56 (1.33-1.79) | 1.48 (1.25-1.71) | 1.37 (1.16-1.57) | 1.51 (1.24-1.78) | 1.42 (1.19-1.65) | 1.60 (1.30-1.90) | 1.47 (1.24-1.70) |
| Nighttime sleep (hours) | 9.24 (8.97-9.51) | 9.30 (9.05-9.55) | 9.38 (9.12-9.65) | 9.44 (9.22-9.65) | 9.20 (8.94-9.47) | 9.38 (9.11-9.65) | 9.53 (9.18-9.88) | 9.63 (9.41-9.86) |
| **Defecation** | | | | | | | | |
| Stool frequency (times/day) | n/a | n/a | 1.14 (1.03-1.25) | 1.16 (1.04-1.29) | n/a | n/a | 1.14 (1.01-1.27) | 1.15 (1.04-1.25) |
| Bristol stool chart, n (%) |  |  |  |  |  |  |  |  |
| Type 1 | n/a | n/a | 4 (2.6) | 4 (5.3) | n/a | n/a | 1 (0.7) | 1 (1.4) |
| Type 2 | n/a | n/a | 16 (10.5) | 12 (16) | n/a | n/a | 10 (6.8) | 9 (12.7) |
| Type 3 | n/a | n/a | 55 (36.2) | 26 (34.7) | n/a | n/a | 63 (42.9) | 32 (45.1) |
| Type 4 | n/a | n/a | 61 (40.1) | 31 (41.3) | n/a | n/a | 53 (36.1) | 26 (36.6) |
| Type 5 | n/a | n/a | 11 (7.2) | 1 (1.3) | n/a | n/a | 8 (5.4) | 0 (0) |
| Type 6 | n/a | n/a | 5 (3.3) | 1 (1.3) | n/a | n/a | 12 (8.2) | 1 (4.2) |
| Type 7 | n/a | n/a | 0 (0) | 0 (0) | n/a | n/a | 0 (0) | 0 (0) |
| **Infection n (%)** | | | | | | | | |
| Respiratory tract infection | 19 (25) | 23 (29.9) | 33 (44) | 27 (35.1) | 18 (25) | 27 (36) | 31 (43.1) | 32 (42.1) |
| Gastrointestinal infection | 2 (2.6) | 3 (3.9) | 2 (2.7) | 2 (2.6) | 0 (0) | 3 (4) | 3 (4.2) | 8 (10.5) |
| Others^2^ | 5 (6.6) | 3 (3.9) | 4 (5.3) | 3 (3.9) | 2 (2.8) | 3 (4) | 2 (2.8) | 2 (2.6) |

Abbreviations: DC, dietary counselling; GI, gastrointestinal; ONS, oral nutritional supplement

^1^ Continuous and categorical data were expressed mean (95%CI) and n (%), respectively. There was no statistically significant difference between DC vs. ONS groups in any of the parameters. Differences in mean and proportion were tested by independent samples t-test and Chi-square test, respectively.

^2^ The number of other infections observed were as follows: Day 15, Fever, unspecified (2), Hand-foot-mouth disease (2), Urinary tract infection(2), Infected wound (1), Cough, unspecified (1) ; Day 30, Fever, unspecified (1), Covid-19 (1), Hand-foot-mouth disease (3), Herpangina (1), Tonsillitis (1); Day 60, Fever, unspecified (2), Urinary tract infection (2), Pneumonia (1); Day 90, Fever, unspecified (1), Hand-foot-mouth disease (1), Influenza infection (1), Dengue infection (1).
